# Integrating cinemeducation and entrepreneurship for experiential learning in public health

**DOI:** 10.1186/s12909-026-09329-x

**Published:** 2026-05-06

**Authors:** Saurabh Parmar, Labhita Das, Damini Joshi, Parthasarathi Ganguly, Anuradha Joshi, Aakash Pareek, Urva Vasavada

**Affiliations:** 1 Parul Institute of Public Health, Parul University, Vadodara, India; 2 Department of Community Medicine, Parul Institute of Medical Sciences and Research, Parul University, Vadodara, India; 3 Parul Institute of Public Health, Parul University, Vadodara, India; 4 Department of Pharmacology, Parul Institute of Medical Sciences and Research, Parul University, Vadodara, India

**Keywords:** Cinemeducation, Critical thinking, Entrepreneurship, Experiential learning, Innovation, Menstrual hygiene, Public health education

## Abstract

**Background:**

Traditional lecture-based teaching methods often fail to stimulate curiosity, critical thinking, and reflective learning among public health students. To address these gaps, a blended pedagogical approach was implemented, integrating cinemeducation, entrepreneurship orientation by the University’s Innovation and Entrepreneurship Research Cell, and a field visit to a sanitary pad-making machine manufacturing unit. This approach aimed to enhance learning on menstrual hygiene, promote critical thinking, and expose students to real-world public health innovation.

**Methods:**

An educational intervention was conducted with 40 Master of Public Health students at a Public Health Institute at Gujarat, India. A mixed-methods design was used, including a structured pre-session orientation, film-based learning, entrepreneurship orientation, and a field visit. Quantitative data were collected through structured Likert-scale questionnaires *(supplementary file 1)* measuring students’ perceptions of learning, critical thinking development, and innovation awareness. Descriptive statistics (mean, SD, 95% CI) were calculated. Qualitative data were obtained from audio-recorded Focus Group Discussions (FGDs), which were transcribed and analyzed using Braun and Clarke’s six-step thematic analysis. Themes were mapped to Kirkpatrick’s Four-Level Evaluation Model to assess educational impact.

**Results:**

Quantitative findings demonstrated high levels of student endorsement of the blended learning approach, with consistently elevated mean Likert scores indicating positive post-intervention perceptions of engagement, critical thinking, and innovation awareness, with mean scores ranging from 4.3 to 4.9 (out of 5) and narrow confidence intervals, indicating strong student endorsement. Qualitative analysis revealed six major themes: (1) Access and affordability of menstrual hygiene products, (2) Cultural stigma and behavioral barriers, (3) Determinants of product choice, (4) The role of government and NGOs, and (5) The educational value of blended learning. (6) Entrepreneurship and Self-Help Groups for Health Solutions Students particularly valued the real-world exposure from the Innovation and Entrepreneurship Research Cell orientation and the manufacturing unit visit, which helped them understand entrepreneurship processes, supply chain challenges, and the socio-economic impact of innovation in public health.

**Supplementary Information:**

The online version contains supplementary material available at 10.1186/s12909-026-09329-x.

## Introduction

The landscape of higher education is undergoing rapid transformation to meet the evolving needs of learners and the global workforce. The traditional lecture-based, one-way classroom teaching is often limited in fostering curiosity, engagement, and deeper learning among learners [1]. Such methods are increasingly inadequate in developing the competencies required for contemporary public health graduates such as critical thinking, problem solving, analytical reasoning, and innovation [2].

Active learning pedagogies such as Problem-Based Learning (PBL) and Team-Based Learning (TBL) have demonstrated to enhance higher-order thinking and clinical reasoning skills as compared to conventional approaches [3, 4]. These methods encourage learners to apply knowledge to real-world situations and foster collaboration and reflection, outcomes that align well with the demands of modern health systems [5]. Umbrella reviews confirm that such student-centered pedagogies contribute significantly to knowledge retention and application in medical education [6].

Innovative approaches such as movie clubs have shown to enhance engagement and reflective learning in psychiatry education [7]. Learners have reported that cinemeducation enhances motivation, contextual understanding, and retention, providing both cognitive and emotional engagement with complex topics [8]. Cinemeducation, defined as the use of films to teach medical and health-related topics, has emerged as a promising method to make learning interactive. Alexander et al. first coined the term “cinemeducation,” emphasizing its value in exploring psychosocial dimensions of health [9]. Films provide a culturally rich medium to explore social determinants of health and patient perspectives, with multiple studies demonstrating its value in medical and health education [10]. Structured film-based sessions can stimulate reflection and critical dialogue, making learning more participatory [11]. Films provide a powerful narrative medium that can trigger emotional engagement, enhance recall, and stimulate critical discussion of complex health issues [12].Cinemeducation​‍​‌‍​‍‌​‍​‌‍​‍‌ has been widely adopted to develop the reflective capacity and empathy of medical and nursing students. However, few studies have investigated the use of this method in public health teaching, especially when it is combined with entrepreneurship and an organized field immersion. The majority of research works reported in the literature have their main focus on the emotional aspects of the learning process and the acquisition of certain skills specific to a particular discipline. They scarcely touch upon the development of systems thinking, innovation, or the application of knowledge for solving problems at the population level, which are the essential competencies of public health ​‍​‌‍​‍‌​‍​‌‍​‍‌professionals [8].

The literature highlights that public health education must evolve to build competencies in leadership, innovation, and entrepreneurship, enabling future professionals to respond to complex health system challenges and explore sustainable solutions [13, 14]. In India, initiatives such as Startup India, Atal Innovation Mission (AIM), and the National Innovation Foundation (NIF) emphasize creativity, innovation, and problem-solving as career pathways for young graduates [15, 16]. Health universities and technology hubs are increasingly aligning their curricula with these national priorities by embedding simulation-based training, incubation centers, and applied research models [17].

The​‍​‌‍​‍‌​‍​‌‍​‍‌ discrepancy between academia and industries is especially noticeable in India, where graduates of public health are anticipated to work in systems that lack resources, challenging socio-cultural environments, and fast-changing innovation ecosystems [14]. Hence,​‍​‌‍​‍‌​‍​‌‍​‍‌ this research implements a blended experiential learning model that fuses cinemeducation, structured entrepreneurship orientation, and real-world field immersion centered on menstrual hygiene to close a significant pedagogical gap. The integration of narrative engagement (film), innovation systems exposure (incubation and entrepreneurship), and contextualized field learning is designed to deepen the learner’s critical reflection, systems thinking, and innovation-related skills, which are of paramount importance to modern public health practice in ​‍​‌‍​‍‌​‍​‌‍​‍‌India [5, 11, 18].

This intervention’s teaching method conceptually matches well with the various adult learning theories. Kolbs Experiential Learning Theory stresses that one learns best by cycling through learning stages: a learner first has a concrete experience, then observes and reflects, then forms concepts and finally tries out the new ideas actively. The cinemeducation session was the time for the learners to have their concrete experience, and the suggested discussions and the visit allowed the learners to reflect and apply the knowledge. Besides, the narrative storytelling and the emotional engagement in the narrative making the students critically question the social taboos around menstrual health and look at ways of the extracurriculum can be solutions is indicative of elements of Transformative Learning Theory. As a whole, the learning theories above pave the way for the blended experiential learning model that was used in the current study.

## Data and methodology

### Study design and setting

This study employed a mixed-methods educational evaluation design using a convergent parallel approach, wherein quantitative and qualitative data were collected during the same intervention period, analyzed separately, and integrated during interpretation to provide complementary insights into learner experiences and perceived educational impact. The study integrated menstrual hygiene education, cultural analysis, and innovation and entrepreneurship exposure into the curriculum for postgraduate public health students. The intervention was implemented in Week 1 and 2 of August 2025 and combined classroom-based and field-based activities.

### Participants

All second-year Master of Public Health (MPH) students enrolled at the Parul Institute of Public Health during the study period were invited to participate in the educational intervention. A total of 40 students consented and participated, representing a complete enumeration of the eligible cohort. Informed consent was obtained, and ethical approval was secured from the Institutional Ethics Committee - Parul University Institutional Ethical Committee for Human Research (PU-IECHR). As this was a blended experiential educational intervention conducted within a predefined academic cohort, a formal sample size calculation was not performed. Instead, all eligible students were included to assess feasibility, acceptability, and perceived educational impact. This approach is consistent with methodological norms in educational and pedagogical research, particularly for exploratory and pilot interventions.

#### Inclusion criteria

1. Were enrolled as second-year Master of Public Health (MPH) students at the Parul Institute of Public Health during the study period.
2. Were present during the intervention period and participated in all core components of the educational activity (cinemeducation session, entrepreneurship orientation, and field visit).
3. Provided written informed consent to participate in the study and associated data collection activities.

#### Exclusion criteria

1. Were absent from one or more components of the intervention.
2. Did not provide informed consent or withdrew consent at any stage of the study.
3. Submitted incomplete quantitative questionnaires or did not participate in the focus group discussions.

### Intervention components and timeline

**Fig. 1:**
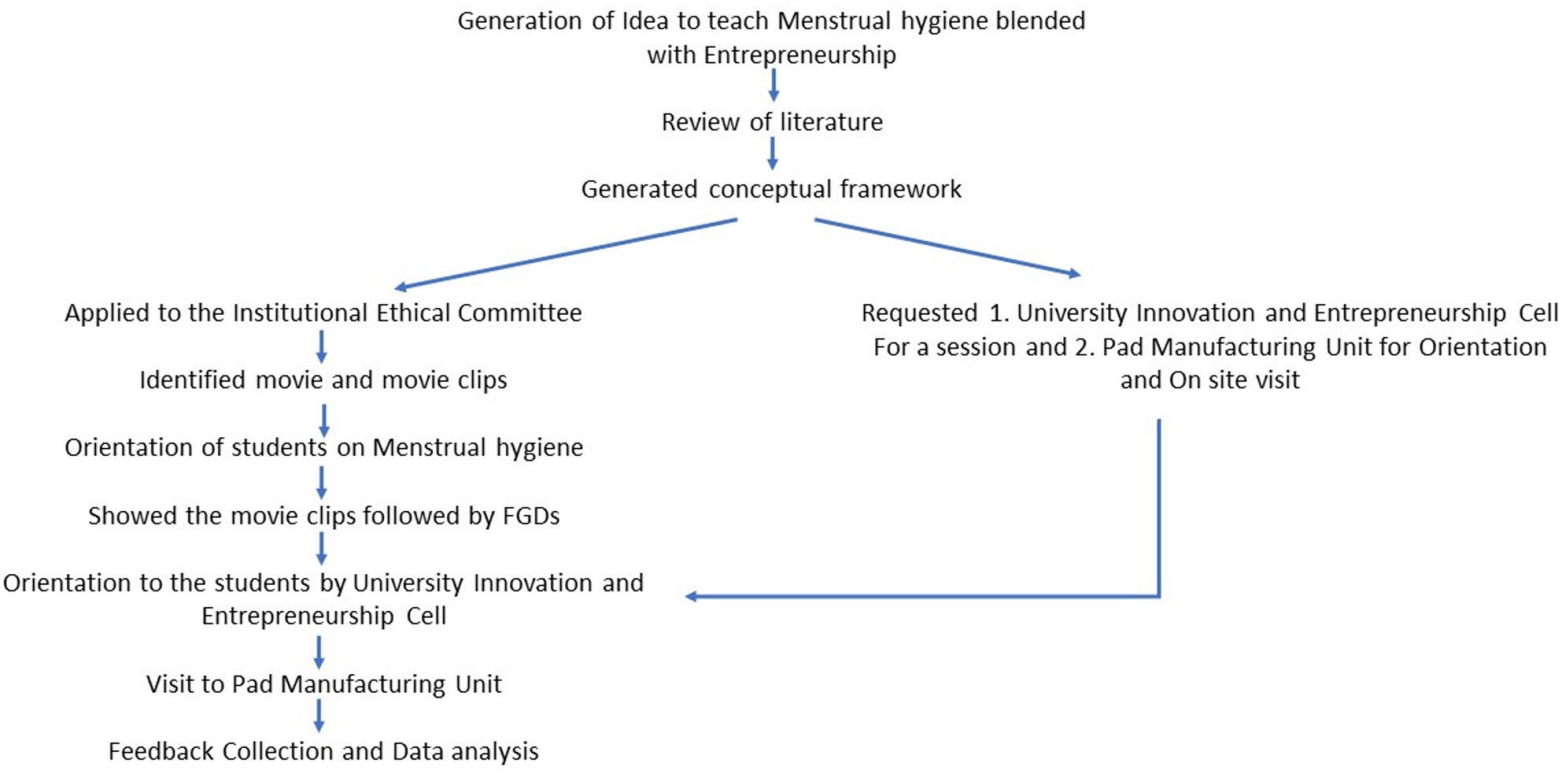
Framework of the methodology

The framework of the methodology is presented in Fig. 1.

#### Week 1: Orientation and cinemeducation

1. Orientation to Menstrual Hygiene: Students attended an interactive session introducing menstrual hygiene, its socio-cultural determinants, factors affecting the product choices, and public health significance.
2. Cinemeducation session using *Padman*: Selected scenes from the Bollywood film *Padman* were screened to illustrate stigma, innovation, access barriers, and economic considerations around menstrual hygiene. The session was facilitated by faculty using guided questions to encourage discussion. Students completed a brief, non-graded pre-session reflective exercise intended to orient them to key concepts rather than to generate analyzable baseline data. A focused group discussion (FGD) was conducted post-screening by dividing the students into 3 groups (Groups 1–13 students, group 2–13 students, group 3–14 students). This FGD was conducted immediately after the cinemeducation session to capture students’ immediate reactions, emotional engagement, and reflective learning specific to the film-based pedagogy, corresponding primarily to Kirkpatrick’s Reaction (Level 1) and Learning (Level 2) outcomes. The FGD was guided by a structured question set to elicit perceptions about affordability, stigma, family dynamics, curiosity, empathy, and problem-solving mindset and innovative thinking. The FGD discussion guide was developed through an iterative process. Initial domains were identified based on the study objectives and a review of literature on cinemeducation, experiential learning, and menstrual hygiene education. Draft questions were prepared to explore perceptions related to stigma, affordability, empathy, innovation, and problem-solving orientation. The guide was reviewed independently by three faculty members with expertise in public health education and qualitative research to ensure content relevance and clarity. Minor revisions were made based on their feedback before field implementation.

#### Week 2: Entrepreneurship and real-world exposure

3. Entrepreneurship orientation: Students attended an interactive session with faculty and incubation managers from the University’s Innovation and Entrepreneurship Research Center. This center fosters research, innovation, and startup culture within the university. The session introduced students to incubation processes, funding strategies, and opportunities for public health entrepreneurship.
4. Field visit to a sanitary pad manufacturing unit: Students visited a local NGO-led enterprise run by a social entrepreneur, specializing in low-cost pad-making machines and biodegradable sanitary pads. During the visit, students observed manufacturing processes, learned about quality control, sustainability challenges, and community engagement strategies. The visit emphasized the linkage between innovation, social entrepreneurship, and public health needs. Following completion of the entrepreneurship orientation and field visit, FGDs were conducted in the same three groups to capture students’ reflections on the overall, integrated impact of the blended intervention. These discussions focused on perceived learning, intended application, and systems-level understanding, corresponding to Kirkpatrick’s Learning (Level 2), Behavior (Level 3), and Results (Level 4).

### Data collection instruments

The Likert-scale questionnaire was designed to capture students’ perceived engagement, relevance, and educational value of the blended pedagogical intervention rather than objective learning outcomes.The item development was guided by the literature concerning cinemeducation, experiential learning, and active learning in health professions education, and was in line with the intervention objectives. Three faculty members with expertise in public health education reviewed draft items to ensure content relevance and clarity.

The questionnaire was also pre-tested with a small group of postgraduate students (*n* = 5) who were not part of the study to assess the clarity and interpretability, and therefore, minor wording changes were made. Due to the exploratory nature of the study and the evaluative purpose of the questionnaire, formal psychometric validation was not performed. The instrument was intended to record the post-intervention perceptions and not as a means to measure objective learning ​‍​‌‍​‍‌​‍​‌‍​‍‌gains. Given the exploratory and formative nature of this educational evaluation, formal psychometric testing such as construct validity assessment or internal consistency reliability estimation was not undertaken. The questionnaire was intended to assess post-intervention perceptions rather than objective learning outcomes.

Each FGD lasted approximately 45–60 min and was facilitated by trained faculty moderators familiar with qualitative methods but not directly involved in student assessment. A note-taker was present during each discussion to document non-verbal cues and group dynamics. Data saturation was considered achieved when no new themes emerged across successive focus groups, and thematic patterns became recurrent across all three groups.

Trustworthiness of the qualitative analysis was enhanced through multiple strategies. Credibility was supported by independent coding by two researchers and consensus discussions. Dependability was ensured through maintenance of an audit trail documenting coding decisions and theme development. Confirmability was strengthened by reflexive memo-writing and regular team discussions to minimize individual interpretive bias.

### Analytical plan

#### Quantitative analysis

For each surveyed domain, descriptive statistics including mean, standard deviation (SD), and 95% confidence interval (CI) were calculated for Likert item scores. Comparative assessments were performed when relevant, with mean Likert scores representing the aggregate student response across domains such as cultural awareness, empathy, and perceived relevance to future public health practice. These statistics were used to quantify central trends and the precision of estimates for post-intervention perceptions following the cinemeducation intervention, aligning with standard practices in educational intervention studies.

#### Qualitative analysis

The qualitative data were analyzed using Braun and Clarke’s six-step reflexive thematic analysis framework: (i) familiarization with data, (ii) generating initial codes, (iii) searching for themes, (iv) reviewing themes, (v) defining and naming themes, and (vi) producing the report. In​‍​‌‍​‍‌​‍​‌‍​‍‌ order to raise the analytic rigor, the coding was done separately by two researchers who had formal training in qualitative methods. The initial codes were compared by iterative consensus meetings, where differences were resolved by discussion and consultation with the original transcripts. The researchers kept reflexivity by means of analytic memos in which they recorded their assumptions and interpretive ​‍​‌‍​‍‌​‍​‌‍​‍‌decisions.

After​‍​‌‍​‍‌​‍​‌‍​‍‌ the themes were finalized, the mapping of themes to Kirkpatrick’s Four-Level Evaluation Model was done. Kirkpatrick’s model served as the interpretive framework to help understand the qualitative findings, not as a tool to measure outcomes directly. In fact, Level 1 (Reaction) and Level 2 (Learning) findings were supported by the participants’ reported experiences and reflections. However, Levels 3 (Behaviour) and 4 (Results) were not subject to empirical measurement as part of this study. We inferred these levels mainly through the cautious reading of participants’ stories, which revealed their potential for application, changes in attitude and perception of the real-world relevance of public health practice. Themes referring to emotional engagement and perceived relevance were considered as Reaction (Level 1); themes showing knowledge acquisition or change of understanding were mapped to Learning (Level 2); themes implying the use of the learning or behavioral intention were connected with Behavior (Level 3); and themes drawing the possible system-level or societal implications were linked with the Results (Level 4). This helped the model to serve as an interpretive analytic framework grounded in the real data, rather than a post-hoc ​‍​‌‍​‍‌​‍​‌‍​‍‌overlay. The Kirkpatrick model was used as an interpretive conceptual framework to organize and contextualize qualitative findings rather than as a formal evaluative instrument measuring behavioral or institutional outcomes.

Findings from both quantitative and qualitative strands were integrated to provide a comprehensive understanding of the intervention’s impact on learners.

### Ethical considerations

The study was approved by the Parul University Institutional Ethical Committee for Human Research (PU-IECHR) (Reference No: ECR/702/Inst/GJ/2015/RR-21/8905). Participation was voluntary, and confidentiality was maintained by anonymizing transcripts and responses. No personal identifiers were collected.

## Results

### Participant profile

A total of 40 MPH learners participated in the cinemeducation intervention. All participants completed the pre-session reflective exercise, film-based session, and focus group discussions. Students represented diverse academic and cultural backgrounds, with 12 male and 28 female participants.

### Thematic analysis

Analysis of the FGD transcripts yielded six overarching themes and several subthemes, derived using Braun and Clarke’s six-step framework (Table 1). These themes captured learners’ reflections on menstrual hygiene determinants, their affective responses to the film, and their learning experience.

**Table 1:** Themes identified through Braun and Clarke’s thematic analysis and their alignment with Kirkpatrick’s Four-Level Evaluation Model

Kirkpatrick Level	What it Captures	Themes Mapped	Illustrative Evidence	Educational Implication
Level 1: Reaction	Learner engagement & perceived relevance	Cultural Norms & Stigma; Affordability & Household Economics	“Shops wrap pads in newspaper”; “Pads as costly as jewelry”; “Felt inspired after seeing local women manufacturing pads”	Cinemed plus real-world exposure can elicit emotional, cultural, and social self-awareness
Level 2: Learning	Knowledge and attitudinal gains	Institutional & Policy Mechanisms; Product Quality & Usability	“School ambassadors as nodal points”; “Reusable pads require hygiene education”; “Local unit produces biodegradable pads with good quality checks”	Strengthen curriculum on policy-practice gaps, culturally responsive health product design, and sustainable innovations
Level 3: Behavior	Intended application or change in practice	Community-Centric Communication; Entrepreneurship & Self-Help Groups	“ASHA should use pads herself before promoting”; “Self-help groups can sustain supply”; “Exposure to entrepreneurs gave us ideas for community-driven business models”	Prepare students to leverage trusted community networks and foster public health entrepreneurship
Level 4: Results	Wider systemic or societal outcomes	All themes	“Mixed NGO-government supply models can improve uptake”; “Branding and local production reduce stigma and cost”; “Local enterprises empower women and reduce dependency on external suppliers”	Long-term: improved menstrual health access, reduced stigma, stronger community ownership, and strengthened local innovation ecosystems

### Access and affordability

Students emphasized the prohibitive costs of commercial sanitary pads and the implications for household budgeting. Several participants reported that affordability was central to sustained adoption of menstrual products, and compared costs with essential household expenses.

### Cultural norms and stigma

Strong cultural influences shaped participants’ perceptions of menstrual hygiene. The wrapping of pads in newspapers or opaque bags, avoidance of men in purchase contexts, and anticipated disapproval from family elders highlighted persistent stigma.

### Determinants of product choice

Discussions revealed that choices were guided not only by cost but also by comfort, availability, and suitability for daily activities. Cloth use persisted due to habit, accessibility, and cultural acceptance.

### Government and NGO roles

Students critically reflected on supply-side barriers, citing examples of policy-practice gaps, procurement delays, and lack of pad distribution through Anganwadi centers. At the same time, they acknowledged NGO contributions and community-based models that provided access.

### Educational value of Cinemeducation

Students described the cinemeducation experience as “memorable,” “interactive,” and “thought-provoking,” noting that it stimulated discussion around multiple determinants of menstrual health.

The themes were mapped to Kirkpatrick’s Four-Level Evaluation Model to align findings with established educational outcomes (Table 1).

### Identified themes & subthemes

- 1. Affordability & Household Economics.
  • Price sensitivity, competing family priorities, perception of pads as a “luxury” item.
- 2. Cultural Norms & Stigma.
  • Menstrual segregation, concealment practices, gendered avoidance, intergenerational enforcement.
- 3. Community-Centric Communication.
  • Trust in local messengers (ASHA workers, peers).
  • Body language, relatability, shared lived experience as acceptance drivers.
- 4. Product Quality & Usability.
  • Fit with lifestyle, comfort, leakage prevention.
  • Reusable options with hygiene education.
- 5. Institutional & Policy Mechanisms.
  • School-based programs (RKSK, Health & Wellness Ambassadors, Gauravi Diwas).
  • Gaps between policy intent and last-mile delivery.
  • Infrastructure issues (vending machines, menstrual corners).
- 6. Entrepreneurship & Self-Help Groups for Health Solutions.
  • Grassroots innovation, cost-effective manufacturing, branding and marketing.
  • Group-based microfinance and production models.

**Fig. 2:**
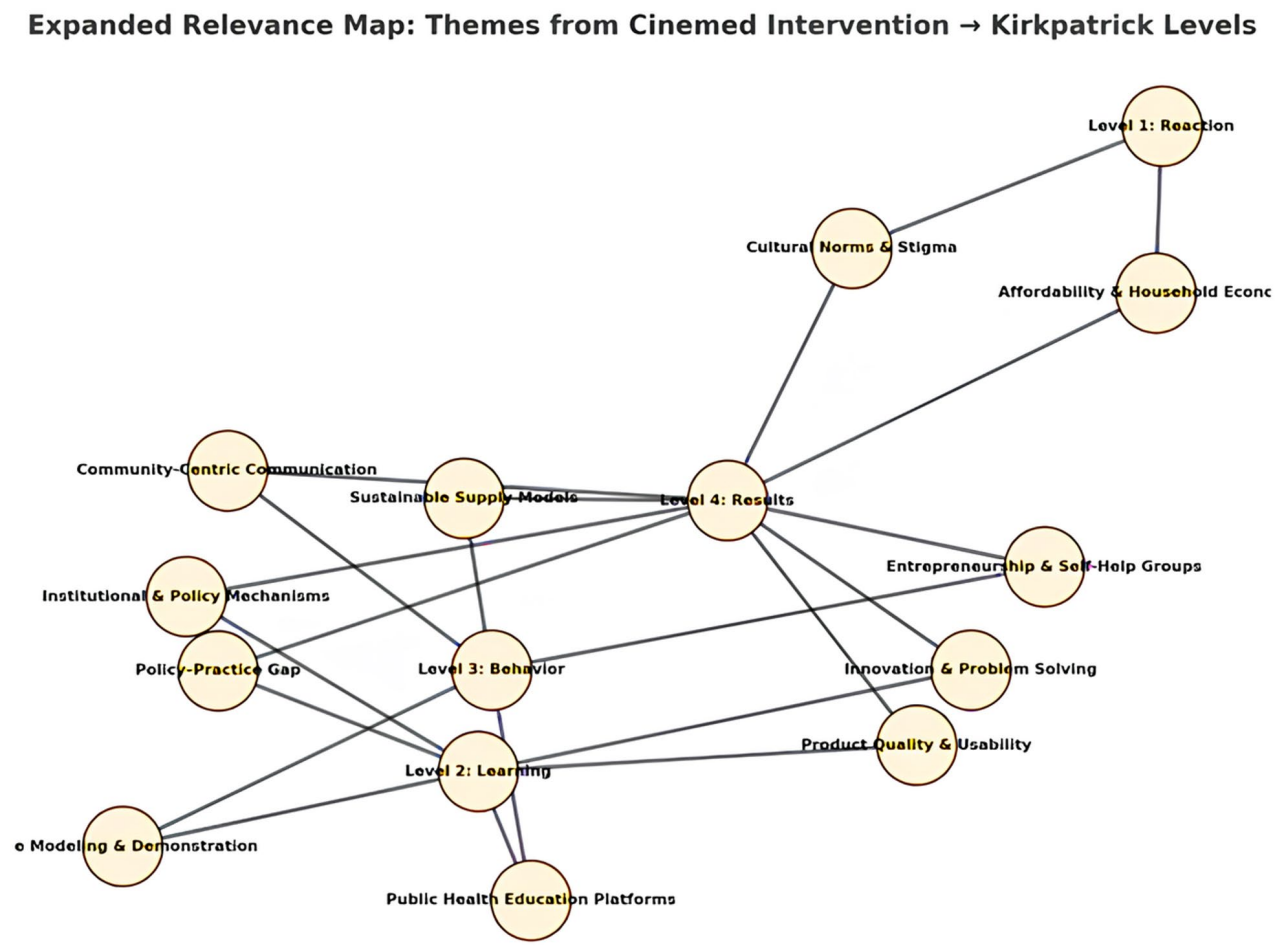
Expanded relevance map: themes from cinemed intervention⇒ Kirkpatrick Levels

Figure 2 presents a conceptual relevance map illustrating the alignment between identified qualitative themes and Kirkpatrick’s four levels of educational outcomes (Fig. 2).

#### Mapping to Kirkpatrick’s Model

The qualitative themes were mapped to Kirkpatrick’s Four-Level Evaluation Model to conceptually situate learner responses across levels of reaction, learning, post-intervention perceptions, and perceived application. This mapping was intended as an interpretive framework rather than a quantitative classification, illustrating how students’ reflections extended beyond immediate engagement to deeper learning and perceived relevance for practice.This mapping is an interpretive alignment based on qualitative insights rather than a direct measurement of behavioural or system-level outcomes, so it is important to note that.

Each node represents either an analytical theme or an educational outcome level, and each edge represents the strength of relevance between the two. The thematic visual map shows how close different themes are that were identified based on their conceptual relevance and co, occurrence in student discussions. The themes that are closer to each other represent the strongest connection that the students perceived, while the ones in the centre indicate the most prominent themes throughout the dataset. The illustration of student comments when superimposed on Kirkpatricks model reveals the learners progression from the first level of engagement and emotional reaction to deeper learning, attitudinal reflection, and perceived relevance for public health practice. This figure serves as a conceptual tool to help in understanding the qualitative results rather than being a quantitative representation (Fig. 2).

### Visual mapping

The relationships between themes and educational outcomes were further depicted in a relevance map (Fig. 2). Proximity of nodes indicated strength of conceptual linkage. For example, Cultural Norms & Stigma clustered closer to Reaction, while Government and NGO Roles aligned with Behavior and Cinemeducation’s Educational Value extended toward Results.

Following thematic analysis, a keyword frequency analysis was performed as a secondary, descriptive procedure to provide additional support for the interpretation of the dominant concepts within the qualitative dataset. The keywords were not taken directly from the raw transcripts but were derived systematically from the finalized codebook.

In particular, the researchers looked through the code labels and the data extracts associated with them to find the words and phrases that were repeated and that best represented the central ideas of each theme (e.g., affordability, stigma, innovation, entrepreneurship). To avoid duplications, synonymous words and closely related expressions were merged into one unified keyword category.

The frequency of each keyword category was determined by the number of its occurrences in individual participant responses. Thus the effect of verbosity of a particular participant is minimized. In this way, keyword frequencies indicate the relative prominence of concepts across the dataset, rather than linguistic repetition.

Keyword analysis was merely another layer of analysis that was used to support and illustrate the thematic findings visually and descriptively and not as a separate method of inference.

Table 2 presents keyword frequencies derived from coded qualitative data, serving as a descriptive complement to the thematic analysis by highlighting the relative prominence of key concepts across participant responses.

**Table 2:** Frequency of key concepts derived from coded qualitative data across categorical domains (*n* = 40)

Rank	Keyword	Frequency	Percentage
Category 1: Menstrual hygiene is recognized as a public health issue			
1	Menstrual health (menstrual, sanitary)	40	100
2	Hygiene practices (hygiene, products)	29	72.5
3	Community involvement (community, schools)	28	70
4	Awareness creation (awareness, educate, education)	28	70
5	Access to products (sanitary pads, menstrual products)	19	47.5
6	Health promotion (health)	16	40
Category 2: Why teamwork is important in Public Health?			
1	Health outcomes (health, better)	40	100
2	Diversity of skills (diverse, different)	31	77.5
3	Teamwork and collaboration (teamwork, team, work)	29	72.5
4	Public health impact (public, community)	24	60
5	Addressing complex issues (complex)	17	42.5
Category 3: Overall feedback on activity			
1	Relevance to health/public health (health, public, issues)	37	92.5
2	Activity engagement (activity, way, learn)	24	60
3	Positive experience (great, good)	24	60
4	Constructive reflection (would, think)	14	35

Frequencies represent the number of participants referencing each concept at least once. Percentages are calculated using the total sample size

Based on the summary provided in Table 2, the patterns of keyword frequency have been instrumental in the thematic findings by showing the hierarchies of the key concepts in different domains. The textual format, instead of repeating the results in the table, concentrates on the interpretive insights that were obtained from these patterns, to demonstrate how the students emphasized menstrual health education, collaborative practice, and the perceived relevance of the blended learning approach.

### Quantitative findings

The quantitative results are presented in Tables 2 and 3. Across domains, students reported consistently high levels of agreement regarding engagement, relevance, and perceived learning value of the blended pedagogical intervention.

**Table 3:** Mean scores, standard deviations (SD), and 95% confidence intervals (CI) for Likert-scale items assessing perceived learning outcomes (1 = Strongly Disagree; 5 = Strongly Agree)

Item (shortened)	Mean	SD	95% CI Lower	95% CI Upper
Q1. I learnt that shame or embarrassment prevents seeking health solutions	4.4	0.7	4.2	4.6
Q2. I realized that films make public health topics engaging	4.2	0.8	4.0	4.4
Q3. The films can effectively illustrate complex concepts	4.8	0.5	4.6	4.9
Q4. The films help in understanding the human aspects of public health	4.4	0.8	4.2	4.6
Q5.The films deepen understanding of health issues	4.6	0.7	4.4	4.7
Q6. Entrepreneurship is a viable career option in public health.	4.2	1.0	4.0	4.5
Q7. I gained awareness of resources supporting public health entrepreneurs.	4.6	0.6	4.4	4.7
Q8. The unit visit showed how innovation addresses public health challenges.	4.4	0.7	4.3	4.6
Q9. The unit visit highlighted real challenges faced by social entrepreneurs.	4.3	0.8	4.1	4.5
Q10. I now understand that public health problems can be solved with creative and innovative approaches.	4.7	0.5	4.6	4.8

### Integration of quantitative and qualitative findings

Triangulation of findings revealed convergence between quantitative and qualitative data. High mean Likert scores indicating perceived engagement, relevance, and innovation awareness were consistent with qualitative themes highlighting emotional engagement, reflective learning, and exposure to real-world problem solving. Quantitative endorsement of entrepreneurship awareness aligned with qualitative narratives describing increased understanding of innovation pathways and social enterprise models.

## Discussion

This study contributes to the growing scholarship on public health pedagogy by demonstrating how cinemeducation, when deliberately integrated with entrepreneurship orientation and real-world field immersion, can function as a cohesive experiential learning model rather than as isolated instructional strategies. The combined approach did not simply function as a sum of separate parts, but rather it seemed to have a synergistic effect by addressing cognitive, affective, and experiential domains of learning, which are usually less engaged in lecture, based formats. Consistent with prior literature on cinemeducation, the narrative structure of film stimulated emotional engagement and facilitated reflective dialogue, enabling students to explore complex social determinants of menstrual health within a culturally contextualized learning environment [12, 19].

The entrepreneurship orientation component was the element that resonated strongly because it spoke about the gap which is very often observed in public health training. It is very rare to show different ways on how to take an idea and make a solution which people can actually use. By positioning innovation within a university, based ecosystem, students could not only think of health problems as issues of policy or service delivery but also as potential social entrepreneurship ventures. This change in the way of thinking is in line with the general changes that are taking place in health education which focus more on the development of skills like leadership, systems thinking, and innovation that are necessary to work in today’s health systems [5, 18, 20].

Likewise, the on-site visit supported the learning process as it helped students to understand the concepts that they had only learned in theory by seeing the actual operations in the real world. Students visiting a women-led sanitary pad manufacturing unit could understand how the factors such as production constraints, affordability, gender empowerment, and community health impact are interrelated. By such a deeply engaging experience, students’ systems thinking as well as their empathy skills were probably developed further since the social and economic aspects of health interventions became more tangible, which is one of the widely acknowledged mechanisms in the experiential learning literature [21]. Besides, this intervention goes well with the principles of Situated Learning whereby knowledge is built in real contexts and through social interaction. Getting in touch with real-world places like a pad manufacturing unit and being part of innovation ecosystems helped learners place theoretical knowledge in community and practice-based settings. This type of involvement might the development of a community-minded view as well as a greater recognition of public health issues as socially embedded phenomena.

This blended intervention design aligns well conceptually with Kolb’s Experiential Learning Theory that views learning as a cycle of four stages: concrete experience, reflective observation, abstract conceptualization, and active experimentation. The cinemeducation segment offered a tangible and emotionally charged experience, which was then followed by student reflection through dialogue. The entrepreneurship orientation and field immersion are additional components of this design that made it possible for students to conduct an abstract thought and gradually build the new concept linking it to the real world. Such correspondence indicates how carefully constructed experiential elements can bring about more profound learning in public health education. Together, these theoretical perspectives provide a coherent framework for understanding how blended, experience-driven pedagogies can support meaningful engagement and contextual learning in public health education.

Unlike prior cinemeducation studies that primarily emphasize emotional engagement or reflective learning within classroom settings, this study extends the pedagogical scope by embedding film-based reflection within an innovation ecosystem and a field-based social enterprise context. This integration enables learners to connect affective insights with systems thinking, implementation realities, and entrepreneurial problem-solving - an area that remains underexplored in public health education literature, particularly in low- and middle-income country contexts.

Also,​‍​‌‍​‍‌​‍​‌‍​‍‌ the business social science aspect dealt with the most significant skill gap in the Indian public health sector educational field, which was very inadequately addressed - lack of access to innovation pathways, financing mechanisms, and implementation realities. Students started to see themselves not just as people who implement programs but also as those who can design solutions and innovate the system at a higher level when public health issues were placed in the context of the entrepreneurial ​‍​‌‍​‍‌​‍​‌‍​‍‌ecosystems.

Collectively, the results highlight that participants perceived film-based learning as a valuable and effective tool for enhancing understanding of public health concepts. The high mean scores across items, combined with the consistency in responses, suggest strong acceptance of this pedagogical approach within the study population. Nonetheless, these interpretations especially for Levels 3 and 4are inferential and exploratory. The study design did not include longitudinal or objective measures to assess behavioural change or real-world impact.

Through team-based learning, these effects were stretched even further, as it offered a collaborative environment for discussion, interpretation, and reflection. Group work enabled participants to deal with different viewpoints, explain their reasoning, and jointly develop their understanding activities that have been recognized as resulting in higher levels of engagement, motivation, and applied reasoning as compared to passive instructional approaches.

The​‍​‌‍​‍‌​‍​‌‍​‍‌ combination of film-based reflection, exposure to innovation systems, and field immersion created a pedagogical synergythat is in line with the current demands of competency-based, systems-oriented public health education. Instead of regarding innovation and empathy as mere secondary skills, this model integrates them into the experiential learning processes which are the most faithful reproduction of the public health practice in the real ​‍​‌‍​‍‌​‍​‌‍​‍‌world.

### Strength and limitations

A key strength lies in the methodological integration of thematic analysis with educational evaluation, offering both depth and impact orientation. Several enabling conditions are critical for other public health institutions to replicate the success of the model. Faculty members who introduce the cinemeducation method must be skilled in facilitating guided discussion and reflective pedagogy so that they can effectively use narrative content for meaningful learning outcomes. Campus support services like innovation or entrepreneurship hubs are capable of taking a step further the learning experience by illustrating the theoretical discussions through real, world innovation ecosystems. Besides, getting hold of suitable material for the films as well as paying due attention to the ethical aspect in the representation of social issues that are sensitive, such as gender norms and menstrual stigma, are amongst the prerequisites for respectful and contextually appropriate class discussions.

This study has several limitations. Without a pre-post or comparator design, it is difficult to draw a causal link between learning gains, and the results are limited to perceived outcomes only, whereas the change in competency has not been measured objectively. Although a brief pre-session orientation exercise was conducted, the study did not employ a formal pre–post test design. Consequently, changes in knowledge or competency could not be quantitatively measured, and findings are limited to post-intervention perceptions. The study conducted in one institution only and the participation being voluntary might have led to a selection bias and, small sample size may limit generalizability. Besides, the use of Kirkpatrick’s higher evaluation levels (Behaviour and Results) relied on inferred qualitative insights and not on direct measurement, which means that it is not possible to draw definite conclusions on long-term impact. Moreover, the focus group discussions on menstrual hygiene-a sensitive topic in terms of culture, could have been affected by the participants’ desire to give socially acceptable answers, which in turn could have influenced the extent or the direction of their responses. The follow-up of the learning and the behavioral change have not been checked here and, therefore, they require a future longitudinal ​‍​‌‍​‍‌​‍​‌‍​‍‌study.

The blended pedagogy described here suggests a promising pathway to enrich public health education. By weaving together film, innovation orientation, and experiential exposure, educators can inspire critical insight and entrepreneurial thinking skills vital for nurturing future public health leaders.

## Conclusion

This paper offers a blended teaching strategy that combines cinemeducation, entrepreneurial orientation, and field immersion to get Master of Public Health students working on menstrual hygiene as a complex nature of the issue. The results indicate that the students liked the intervention, and it helped them reflect on social, cultural, and economic factors as well as giving them a glimpse into innovation and problem-solving in real-life situations.

Both qualitative and quantitative data show that most students thought the intervention was engaging, relevant, and helped them learn through the experience. The thematic analysis brought to light that the use of storytelling in teaching and going to the field were instrumental in developing understanding of the context and compassion. Nevertheless, due to single-group and post-intervention design besides self-reported views, results should be considered more as exposing the learners experiences rather than proving learning gains or behaviour changes.

This paper is a piece of work that adds to the literature on hands-on and innovation-oriented public health education literature by showing that the blended model is implementable and acceptable in an Indian setting. Future studies should include prepost, comparison, and a follow-up after a quite a while to check for impact on competencies and practice more thoroughly.

## Supplementary information

Supplementary Material 1.

## Data Availability

The datasets used and/or analysed during the current study are available from the corresponding author on reasonable request.
