# Supplementary material for: Integrating cinemeducation and entrepreneurship for experiential learning in public health
Source: BMC Med Educ. 2026 May 6;26:909. doi: 10.1186/s12909-026-09329-x (PMC13235040; doi:10.1186/s12909-026-09329-x)
Supplement: Supplementary file 1 — Supplementary Material 1. [file 12909_2026_9329_MOESM1_ESM.pdf]

Questionnaire: Menstrual Hygiene, Innovation, and Public Health Education

Section 1: Informed Consent and Ethics

Consent Statement:
This study adheres to the ethical standards set forth in the Declaration of Helsinki (2013). Ethical clearance was obtained from the Parul University Institutional Ethical Committee for Human Research (PU-IECHR Reference No: ECR702InstGJ2015RR-218905, July 25, 2025). All responses are confidential and anonymized; participation is voluntary, and you may withdraw at any time. No personal identifiers will be collected.
Participant Consent:

- I have read and understood the information above.
- I voluntarily agree to participate in this study.

(Proceeding indicates written electronic consent.)

Section 2: Demographic Information

- Age:
- Gender: [Male] [Female] [Other]
- Academic Background: ____________________
- Prior exposure to public health courses? [Yes/No]

Section 3: Quantitative Survey (Likert Scale: 1 - Strongly Disagree, 5 - Strongly Agree)

No.	Survey Statement	1	2	3	4	5
Q1	Shame or embarrassment prevents seeking health solutions					
Q2	Films make public health topics engaging					
Q3	Films effectively illustrate complex concepts					
Q4	Films help understand human aspects of public health					
Q5	Films deepen understanding of health issues					
Q6	Entrepreneurship is a viable career option in public health					
Q7	I gained awareness of resources for public health entrepreneurs					

Q8	The unit visit showed how innovation addresses public health challenges					
Q9	The unit visit highlighted challenges faced by social entrepreneurs					
Q10	Public health problems can be solved creatively and innovatively					

Section 4: Qualitative Questions (Open-Ended)

I. Social Rules, Stigma, and Menstrual Health

- What challenges do women face due to menstrual isolation in your community?
- How does the cost of sanitary pads affect women's choices and health risks?
- Which factors make it difficult to access menstrual hygiene products in rural areas?
- What role should governments and health organizations play in making health products affordable?

II. Problem-Solving and Entrepreneurship

- What motivated efforts to create or adopt new menstrual hygiene solutions in your experience?
- What skills are most helpful for public health innovation?
- How can public health professionals encourage adoption of new health behaviors?

III. Societal Paradoxes and Cultural Practices

- How do celebrations of menarche contrast with menstrual isolation practices?
- What impact does menstrual isolation have on girls' physical and emotional health?

IV. Communication and Teamwork

- Why is the messenger (e.g., ASHA worker, peer, NGO) important in delivering health solutions?
- What qualities make public health communication effective in sensitive subjects?

V. Experiential Learning Value

- How has film-based education or field visits enhanced your understanding of menstrual hygiene issues?
- What is your feedback on combining entrepreneurship orientation and experiential learning?

Section 5: Thematic Domains (Mapping)

Theme	Example Subthemes
Affordability	Price sensitivity, family priorities
Cultural Norms	Menstrual segregation, stigma, concealment practices
Product Quality	Comfort, usability, leakage prevention
Institutional Policy Mechanisms	School-based programs, policy-practice gaps
Community-Centric Communication	Trust in local messengers, ASHA workers
Entrepreneurship	Grassroots innovation, cost-effective manufacturing

Section 6: Ethics and Data Handling

- All data will be anonymized and stored securely.
- Findings may be published in anonymous aggregate form.
- For further queries, contact: Principal Investigator/Corresponding Author.
